# Pangola grass as forage for ruminant animals: a review

**DOI:** 10.1186/2193-1801-2-604

**Published:** 2013-11-12

**Authors:** Kanitta Tikam, Chirawat Phatsara, Choke Mikled, Therdchai Vearasilp, Wirapon Phunphiphat, Jeerasak Chobtang, Anusorn Cherdthong, Karl-Heinz Südekum

**Affiliations:** Institute of Animal Science, University of Bonn, Endenicher Allee 15, 53115 Bonn, Germany; Department of Animal and Aquatic Science, Chiang Mai University, Chiang Mai, 50200 Thailand; Lampang Animal Nutrition Research and Development Center, Lampang, 52190 Thailand; Bureau of Animal Nutrition Development, Ratchathewi, Bangkok, 10400 Thailand; Tropical Feed Resources Research and Development Center (TROFREC), Department of Animal Science, Faculty of Agriculture, Khon Kaen University, Khon Kaen, 40002 Thailand

**Keywords:** Pangola grass, Tropical forage, Ruminants, Thailand

## Abstract

This review focuses on the introduction and investigation of pangola grass as a tropical forage species especially in Thailand. Pangola grass (*Digitaria eriantha* Steud., synonym *D. decumbens*) is one of recent examples of grasses that have been successfully introduced to Southeast Asia and is often considered as one of the highest quality tropical grasses popularly grown as pasture. Pangola grass is utilized extensively as grass for animal grazing, hay and silage making. Its crude protein content is commonly in the order of 5 to 14% of dry matter and may exceed 15% of dry matter with young regrowth under high fertilization. It has been documented that the type and number of ruminants receiving pangola grass can determine the success of its use. Results obtained when pangola grass in fresh, hay or silage form was fed to ruminant animals as supplements showed better performances in body weight gain, feed conversion ratio, carcass yield, meat quality, and milk yield and composition. In conclusion, pangola grass is a promising forage and a source of high quality feed for ruminant animals in tropical countries.

## Introduction

In several developing countries, ruminant animals are the major contributors to draught power and are increasingly important as a source of meat, milk, and other livestock products. Livestock contribute 10 to 45% to the gross domestic product (GDP) in the developing world, and this contribution is higher if the value of draught power is included in the calculation. It is one of the fastest growing sub-sectors in agriculture (World Bank 2009). Indonesia, Myanmar and Thailand have the largest ruminant populations in Southeast Asia. The livestock numbers continue to increase throughout Southeast Asia despite the increasing human population density in these regions.

Thailand, which lies between 5°30' and 20°30'N and 98° and 105°E, has approximately 0.6 million dairy cattle, 6.5 million beef cattle and 1.2 million buffalo; about 3.4 million families raise these animals on 184,400 ha of forage (Department of Livestock Development 2011), mostly on natural pastures and crop residues.

The common problem of the farmers is the scarcity of good quality forage and the sometimes very high prices during the dry season. Grass and legume pastures are generally sources of green forage for beef cattle during wet or rainy seasons. During the dry season, grasses and legumes stop growing so the farmers need to find alternative roughages for their animals. One way to overcome this problem and to maintain the continuity of feed supply is to conserve surplus forage or crops as hay or silage for later use when feed is in short supply.

Pangola (*Digitaria eriantha* Steud., synonym *D. decumbens*; family: *Poaceae* (alternatively *Gramineae*), subfamily: *Panicoideae*, tribe: *Paniceae*) is one of the highest quality tropical grasses (Cook et al. 2005). It is utilized extensively for grazing, hay or silage making (Meeske et al. 1999), mostly with nitrogen fertilization rather than a companion legume. Therefore, this review focuses on pangola grass with regard to description of its origin and distribution, historical highlights in Thailand, chemical composition, nutritive values and utilization by ruminant animals.

### Description of pangola

Common names for pangola are pangola grass (American and Australian English and Thai), finger grass, digit grass, woolly finger grass (English), digitaria (French), pangolagras (German), pasto pangola (Spanish), pangola digit grass (Florida). Pangola is a stoloniferous perennial and when established, it spreads rapidly by stolons. It does not produce viable seeds. Stems are up to 120 cm high. The leaves are linear-lanceolate to linear, 10 to 25 cm long and 2 to 7 mm wide. The inflorescence has one to two whorls with 5 to 10 spikes that are up to 13 cm long each, with many spikelets 2.70 to 3.00 mm long (Bogdan 1977). Optimal growth conditions are annual precipitations ranging from 700 to 4000 mm, temperatures from 15.9 to 27.8°C and soil pH from 4.30 to 8.50 (Duke 1983), indicating adaptability to a wide range of environmental conditions.

### Origin and distribution of pangola

Pangola is a native of tropical South Africa, thought to originate in the Pongola River in the eastern Transvaal in South Africa or in the adjacent Zululand districts. Pangola is thus thought to be an alteration of the river’s name. The grass was introduced into the United States in 1935 and released for cultivation in 1940. By 1955 there were 202,343 hectares established in Florida, all derived by vegetative reproduction from the two or three plants introduced in 1935. Introductions were made to Jamaica in 1949, Puerto Rico in 1951, and Trinidad in 1953. Since that time it has been introduced widely to Central- and South-America, Australia, West Africa, the Pacific Islands and tropical Asia (Nestel and Creek 1962). Pangola is recommended for the poorly drained soils in Malaysia and the Philippines and is tolerant of flooding (Hacker 1992).

### Historical highlights of pangola in Thailand

Pangola was adapted from the Philippines to areas of Thailand by the Animal Nutrition Division in 1983, and released for cultivation at Pakchong Animal Nutrition Center (which later changed its name to Nakhonratchasima Animal Nutrition Research and Development Center) and redistributed to all areas in Thailand. Until 1992, Dr. T. Yu (at that time at Charoen Pokphand Foods PCL-Crop Integration Business CP Group) imported pangola grass type 254A from Taiwan and cultivated it at Kamphaeng Phet province (Northern Thailand) to produce pangola hay for sale in Thailand and abroad. Pangola grass was introduced to the farmers not until 1999 (Animal Nutrition Division 2006).

In 2002, the Thai government promoted forage production and supported farmers who produced hay and silage instead of rice and other regular cash crops. Pangola grass cultivation replaced rice cultivation in the lowland and was called “Paddy pasture”. The project was called the “Na Yaa” project in Thai. Pangola grass cultivation has now been introduced into all regions in Thailand. Farmers are now planting and using pangola widely as a crop for raising animals. Pangola is being promoted as a high quality fresh grass cash crop for cultivation on former rice lands (Khemsawat and Phonbumrung 2002).

### Chemical composition and nutritive value of pangola

Pangola grass, Para (*Brachiaria mutica*), Ruzi (*Brachiaria ruziziensis*), Purple guinea (*Panicum maximum* TD 58), Napier (*Pennisetum purpureum*), Atratum (*Paspalum atratum)*, Mulato (*Brachiaria hybrid* cv. Mulato), Hamata stylo (*Stylosanthes hamata* cv.Verano), Stylo 184 (*Stylosanthes guianensis* CIAT 184), Cavalcade centurion (*Centrosema pascuorum* cv. Cavalcade), leucaena (*Leucaena leucocephala)* and Desmanthus (*Desmanthus virgatus*) have been the most successful grass and legume species and are commonly found in Southeast Asia, especially in Thailand. In the Philippines and Indonesia, leucaena was widely promoted as a livestock feed. Although these grass and legume species are competing with each other, direct comparisons of both agronomic performance and feeding value are missing and are thus encouraged.

### Chemical composition

Crude protein (CP) values of pangola grass are commonly in the range of 5 to 12% of dry matter (DM; Table 1) but may exceed 15% of DM with young regrowth age and intensive fertilization (Heuzé et al. 2011). Common to all tropical grasses, nutritional value and chemical composition of pangola vary with several factors such as differences in stage of cutting, fertilizer, location, climate and environment. The average CP content of pangola (7.9%) is low compared to those of *Pennisetum purpureum* cv. Mott*, P. purpureum*, *Brachiaria humidicola* and *Panicum maximum* cv. Common (Animal Nutrition Division 2004).

**Table 1:** **Chemical composition of pangola grass (% of dry matter unless stated)**

Pangola form and reference	Cutting age (days)	DM (%)	CP	EE	NDF	ADF	ADL
***Fresh***							
Archimède et al. (2000)	42	-	7.2	-	79.0	44.2	7.8
Lee et al. (2000)	45	39.7	6.7	2.1	-	42.8	-
Animal Nutrition Division (2004)	45	31.8	7.9	2.0	63.3	35.7	4.0
Chaichaum et al. (2007)	40-50	21.5	8.1	2.6	61.2	34.1	-
Assoumaya et al. (2007)	42	-	10.7	-	72.5	35.7	6.4
Angthong et al. (2008)	45	24.8	9.5	1.7	66.4	39.8	4.9
Eugène et al. (2010)	42	-	12.0	-	71.6	35.0	-
Chaiwang et al. (2011)	40-45	22.8	5.3	3.4	73.0	37.7	7.3
Fanchone et al. (2012)	35	-	12.0	-	75.6	38.2	-
SD^1^		7.6	2.4	0.7	6.1	3.5	1.6
***Hay***							
Lee et al. (2000)	70	87.1	3.0	2.0	-	46.6	-
Suzuki et al. (2008)	45	90.1	9.5	1.5	74.6	42.3	5.0
Suksathit et al. (2011)	45	85.4	3.1	3.8	71.7	41.7	4.1
Chobtang et al. (2012)	45	88.7	7.0	1.4	69.5	36.6	4.2
Kaewkunya et al. (2013)	84	89.5	4.3	0.8	69.5	35.1	9.9
SD		2.4	2.8	1.1	2.4	4.6	2.8
***Silage***							
Esperance et al. (1980)	42	20.6	8.0	-	-	-	-
Phunphiphat (2004)	45	34.1	13.4	-	-	-	-
Chaichaum et al. (2007)	45	25.0	7.1	2.5	5.9	39.5	-
SD		6.9	3.4	-	-	-	-

^1^Standard deviation. DM, dry matter; CP, crude protein; EE, ether extract; NDF, neutral detergent fibre; ADF, acid detergent fibre; ADL, acid detergent lignin

Many studies have been made on the chemical composition of pangola utilized fresh or preserved as hay or silage (Table 1). If only studies from Thailand are considered, the CP content varied from 5.3 - 7.9, 3.1 - 10.5 and 7.1 - 13.4% of DM, respectively, for fresh, dried (hay) and ensiled pangola. Archimède et al. (2000) have shown that it was mainly the CP content of pangola grass that decreased with maturity. The decrease in CP content between 14 and 28 days of growth represent 70% of the general decrease observed between 14 and 56 days. Moreover, Assoumaya et al. (2007) reported that the 56-day old grasses were characterised by lower CP and higher fibre contents as compared to 21-day old grasses. The CP values of pangola therefore depend on the stage of growth. In Taiwan, Yeh (1990) reported that the average CP content of pangola varied depending on the regrowth interval length – from 10.5% with cutting at 4-week intervals to 8.9% with cutting at 6-week intervals and 8.0% with cutting at 8-week intervals. An attempt was made to assign the observed variation in chemical composition and particularly CP concentration of pangola to specific sources but this attempt failed as most studies lacked sufficient information on factors such as amount of applied nitrogen fertilizer or soil type and fertility. For this reason, a quantitative evaluation of factors contributing to variation in chemical composition of pangola was not possible.

### Nutritive value of pangola and utilization by ruminants

Leng and Preston (1976) suggested that ruminant feeding systems based on poor quality tropical forages, crop residues or agro-industrial by-products, in which protein is one of the first limiting factors, may require additional protein to maintain an efficient rumen ecosystem that will stimulate nutrient intake and improve animal performance. Several authors subsequently showed that pangola could efficiently serve as a protein supplement to such diets. If used as a supplement to low-CP forage or mixed diets based on, e.g., straw, CP concentrations above 15% of DM (see previous section) can be accepted as the greater proportion of non-protein-nitrogen in the CP of intensively fertilized forage can be effectively converted by rumen microbes into microbial amino acids, and finally, microbial protein (review by Leng 1990).

Results of studies involving pangola varied widely depending on the form of its presentation and species of animal. The general conclusion is that supplementation of pangola grass in fresh or preserved (hay and silage) forms to ruminant animals showed beneficial results. Ranchers in Central and South Florida have been well served for many years by ‘pangola’ and other cultivars of digit grass. Pangola is a very palatable grass that is readily consumed by livestock (beef, dairy cattle and horses) as grazed pasture or hay (Vendramini et al. 2012). In the following paragraphs, results are summarized from studies involving *in vivo* and *in vitro* measurements and these are separately presented.

*In vitro* data were only reported by Regan (2000) and Juárez Reyes et al. (2009). Regan (2000) reported that in northern Australia bale silage was prepared from wilted pasture with pangola grass (*Digitaria eriantha* subsp. e*riantha*) and two legumes, namely cavalcade centurion (*Centrosema pascuorum*) and wynn cassia (*Chamaecrista rotundifolia*). The DM content of the silages made from the wilted plants ranged from 42–57%, the *in vitro* digestibility from 55.0-58.0% and the estimated metabolizable energy concentration from 8.5-9.0 MJ/kg DM. Juárez Reyes et al. (2009) reported higher *in vitro* gas production (P < 0.05) for pangola grass, compared with 30% less gas in other grasses (Guinea, Bermuda and Tanzania grasses). They also reported greater (P < 0.05) *in vitro* gas production and insoluble but slowly degradable (b) fraction of pangola grass, as well as lower b fraction in Guinea and Bermuda grasses.

Although a reasonable number of *in vivo* studies have been published, they vary largely in terms of animal species or category within species, research methods and, even more important, response variables studied. This extreme heterogeneity, which left only few and sometimes one single number for a given variable related to the nutritive value of pangola, precluded a quantitative analysis of data. It became obvious that more systematic and comparative studies are needed before this goal can be achieved.

Lee et al. (1991) indicated that the digestibility of DM and DM constituents and hence the energy value of pangola and Napier grasses were higher at the earlier stages of growth and decreased as the plant approached maturity. More specifically, Archimède et al. (2000) reported that OM digestibility decreased curvilinearly with age. Seventy-one percent of the total decrease occurred between 14 and 28 days with the corresponding values for neutral detergent fibre and acid detergent fibre of 75 and 69%. More recent studies on *in vivo* digestibility of pangola fed in different forms are summarized in Table 2. This data again suggest a considerable variation even for pangola harvested after similar or the same length of the growth period but, as already stated for chemical composition, data do not allow to clearly identifying the reason for this variation. Panjaitan et al. (2010) fed Spear and Mitchell grass hays (low quality tropical forage) to steers and observed lower microbial CP (MCP) production (80 and 170 g MCP/day, respectively) and efficiency of MCP production (78 and 79 g MCP/kg digestible organic matter (DOM), respectively) than when steers were fed pangola grass (328 g MCP/day; 102 g MCP/kg DOM) and ryegrass (627 g MCP/day; 135 g MCP/kg DOM) hays, which was directly related to the supply of DOM and rumen-degradable CP.

**Table 2:** **In vivo nutrient digestibility of pangola (%)**

Pangola form and reference	Cutting age (days)	Type of animal	DM	OM	CP	NDF	ADF
***Fresh***							
Lee et al. (2000)	45	Dairy goats (n = 6)	79.8	-	61.9	-	-
Mullik et al. (2009)	-	Brahman steers (n = 4)	59.7	68.6	52.3	69.9	-
Eugène et al. (2010)	42	Black Belly rams (n = 16)	-	64.8	73.7	74.9	-
SD^1^					10.7		-
***Hay***							
Lee et al. (2000)	70	Dairy goats (n = 6)	54.1	-	34.0	-	-
Angthong et al. (2006)	45	Brahman cattle (n = 4)	61.0	64.0	62.0	67.9	64.8
Pitaksinsuk et al. (2007)	45	Brahman cattle (n = 4)	60.3	62.6	57.2	71.4	63.4
Suzuki et al. (2008)	45	Brahman steers (n = 4)	58.1	60.7	53.7	68.0	66.3
Suksathit et al. (2011)	45	Thai native cattle (n = 4)	74.5	76.7	78.9	66.2	54.6
Chobtang et al. (2012)	45	Brahman cattle (n = 4)	55.6	58.5	45.4	56.0	49.9
Kaewkunya et al. (2013)	84	Crossbred lambs (n = 16)	78.5	-	66.2	79.5	73.2
SD			9.5	7.1	14.5	7.6	8.4

^1^Standard deviation. DM, dry matter; OM, organic matter; CP, crude protein; NDF, neutral detergent fibre; ADF, acid detergent fibre.

Some authors studied effects of pangola on growth of ruminants. In Taiwan, Hsieh (1990) used four tropical grasses (pangola, Guinea, dwarf elephant and South African pigeon grass) which were grazed by seven Holstein steers during the first year and 60 Nubian-native goat hybrids during the second year. Pangola gave the highest average daily gain (ADG) for cattle, while Guinea grass was the second highest. At Parada in North Queensland, Australia, a body weight gain of 2,990 kg/(ha × year) was obtained from grazing irrigated pangola grass fertilized with 672 kg N/(ha × year); (Ebersohn and Lee 1972). In Jamaica, Creek and Nestel (1965) found that pangola grazed at 32-day intervals produced more DM and more body weight gain than when grazed at 40-day intervals.

In Thailand, the quality of beef from cattle fed with pangola in terms of chemical composition, collagen content, cholesterol, triglyceride and water holding capacity showed different values between White Lamphun and Brahman crossbred cattle (Chaiwang et al. 2011). However, pangola can be used for rearing native cattle and could be an alternative feed for farmers. Moreover, Tuikampee et al. (2006) reported that the average milk yield of cows fed pangola grass-based diets was approximately 16 kg/day; the milk had 3.51 - 3.61% fat, 2.93 - 2.94% protein, 5.02 - 5.04% lactose and 11.97 - 12.00% total solids. Moreover, Chobtang et al. (2012) reported that methane emissions from bulls that received good quality pangola hay (28-day growth period) were significantly lower (P < 0.05) than those of bulls fed medium quality hay (45-day growth period). These authors postulated that the use of good quality hay can contribute not only to improving nutrient supply to the animal but also to reducing greenhouse gas emission to the atmosphere when compared with lower quality hay.

Ruminal disorder or toxicity was not found in sheep, goat, beef cattle and dairy cattle. However, in horse grazing pangola pastures, cases of bighead disease (nutritional secondary hyperparathyroidism caused by interference of oxalate in pangola grass with mineral utilization in horses) have been recorded (Stewart et al. 2010). In suitable environments, pangola grass – through improved intake and digestibility – can support better ruminant performance in terms of milk yield and composition, body weight gain, feed conversion ratio and meat quality than most other introduced pasture grasses in the Tropics.

## Conclusions

The future of pangola grass as basal roughage or supplement in the diets of ruminant animals seems to be very promising but requires long term plans. Pangola grass is a good source of forage and can be fed fresh or preserved as hay or silage. Thus, pangola is regarded as one of the most digestible and highest quality tropical grasses and expected to be good feed for ruminant animals.
